# Quantitative multiplex immunofluorescence analysis identifies infiltrating PD1^+^CD8^+^ and CD8^+^ T cells as predictive of response to neoadjuvant chemotherapy in breast cancer

**DOI:** 10.1111/1759-7714.13639

**Published:** 2020-09-07

**Authors:** Hongling Liang, Hongsheng Li, Zhi Xie, Tianen Jin, Yu Chen, Zhiyi Lv, Xiaojun Tan, Jia Li, Guodong Han, Weixing He, Ni Qiu, Ming Jiang, Jie Zhou, Haoming Xia, Yongtao Zhan, Lulu Cui, Weiling Guo, Jianqing Huang, Xuchao Zhang, Yi‐long Wu

**Affiliations:** ^1^ The Second School of Clinical Medicine Southern Medical University Guangzhou China; ^2^ Department of Breast Oncology Affiliated Cancer Hospital & Institute of Guangzhou Medical University Guangzhou China; ^3^ Guangdong Lung Cancer Institute, Guangdong Provincial Key Laboratory of Translational Medicine in Lung Cancer Guangdong Provincial People's Hospital, Guangdong Academy of Medical Sciences, School of Medicine, South China University of Technology Guangzhou China; ^4^ Guangzhou DaAn Clinical Laboratory Center Guangzhou China; ^5^ Department of Pathology Affiliated Cancer Hospital & Institute of Guangzhou Medical University Guangzhou China; ^6^ Graduate School of Arts and Science Columbia University in the City of New York New York New York USA

**Keywords:** Breast cancer, neoadjuvant chemotherapy, PD1, T cell, tumor‐infiltrating lymphocytes

## Abstract

**Background:**

This study aimed to explore the potentially predictive role and dynamic changes of immune checkpoints on T cell subsets in patients with breast cancer receiving neoadjuvant chemotherapies.

**Methods:**

Fluorescent multiplex immunohistochemistry (mIHC) was used to stain CD4, CD8, PD1, TIM3, and cytokeratins simultaneously in paired breast cancer samples before and after neoadjuvant therapies (NAT) in a prospective cohort (*n* = 50). Singleplex IHC was conducted to stain for CD3 in 100 cases with inclusion of extra retrospective 50 cases. Cell levels were correlated with clinicopathological parameters and pathological complete response (pCR).

**Results:**

In pretreatment tumors, the percentages of infiltrating CD8^+^, PD1^+^, PD1^+^CD8^+^, and the ratio of PD1^+^CD8^+^/CD8^+^ cells, were higher in pCR than non‐pCR patients in either the stromal or intratumoral area, but PD1^+^CD4^+^, TIM3^+^CD4^+^, TIM3^+^CD8^+^ cells and CD4^+^/CD8^+^ ratio was not. Multivariate analyses showed that the percentage of intratumoral CD8^+^ cells (OR, 1.712; 95% CI: 1.052–2.786; *P* = 0.030) and stromal PD1^+^CD8^+^/CD8^+^ ratio (OR, 1.109; 95% CI: 1.009–1.218; *P* = 0.032) were significantly associated with pCR. Dynamically, reduction in the percentages of PD1^+^, CD8^+^ and PD1^+^CD8^+^ cells after therapy strongly correlated with pCR. Notably, incremental percentages of PD1^+^CD8^+^ cells, rather than TIM3^+^CD8^+^, were shown in tumors from non‐pCR patients after NAT. CD3 staining confirmed the percentage of T cells were associated with pCR.

**Conclusions:**

PD1^+^CD8^+^ rather than TIM3^+^CD8^+^ cells are main predictive components within tumor‐infiltrating T cells in NAT breast cancer patients. Dynamically incremental levels of PD1^+^CD8^+^ cells occurred in non‐pCR cases after NAT, suggesting the combination of chemotherapy with PD1 inhibition might benefit these patients.

**Key points:**

**Significant findings of the study:**

- PD1^+^CD8^+^, rather than TIM3^+^CD8^+^, T cells are the main component to predict the response of neoadjuvant therapies in breast cancer.

**What this study adds:**

- Incremental levels of PD1^+^CD8^+^ T cells in non‐pCR post‐NAT tumors suggest PD1 inhibition might benefit in the neoadjuvant setting.

## Graphical abstract

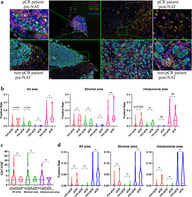

PD1^+^CD8^+^, rather than TIM3^+^CD8^+^, T cells are the main component that could predict the response of neoadjuvant therapies in breast cancer. Incremental levels of PD1^+^CD8^+^ T cells in non‐pCR post‐neoadjuvant‐treatment tumors suggest PD1 inhibition might be beneficial to patients.

## Introduction

The incidence and mortality of breast cancer (BC) remain high,^1^, ^2^, ^3^ despite advances in cancer detection technologies and therapeutic interventions over past decades. The treatment approaches, as well as the prognosis of BC patients, vary depending on the molecular and pathological subtype. For patients diagnosed with early‐stage BC, surgery remains the standard of care. Neoadjuvant therapies (NAT) are also often provided for operable tumors. Complete tumor regression and pathological complete response (pCR) have been associated with improved survival.^4^, ^5^, ^6^ A strong positive association between pCR and improved outcomes in BC patients undergoing NAT already supported the opening of an accelerated drug approval pathway.^7^ However, the accurate prediction of pCR in a clinical setting remains challenging.

Apart from the ER or HER2 status of the primary tumor,^8^ genomic markers^9^, ^10^ and the presence of tumor‐infiltrating lymphocytes (TILs)^11^, ^12^, ^13^ have been reported to affect the likelihood of pCR. In retrospective trial cohorts, TIL levels have recently been identified as an independent predictor of pCR in BC.^14^, ^15^

The role of the different immune cell subtypes in the tumor microenvironment is not fully understood. The tumor microenvironment (TME) is extremely complex, containing different types of lymphocytes, macrophages, and myeloid‐derived suppressor cells (MDSC), etc. In BC, the vast majority of TILs are CD4^+^ T cells, CD8^+^ T cells and B cells.^16^ CD8^+^ T cells are traditionally considered cytotoxic T cells as they are able to kill cancer cells directly. Increased levels of infiltrating CD8^+^ T cells have been reported to be associated with improved survival in BC patients, regardless of the molecular subtype.^17^ Tumor‐infiltrating CD4^+^ or CD8^+^ T cells by tissue microarray‐based immunohistochemistry (IHC) were reported in association with response to neoadjuvant chemotherapy.^18^, ^19^, ^20^ Computational pathology can provide objective, quantitative, and reproducible tissue metrics and represents a viable means of outcome prediction in BC.^21^ However, different methods may have varied accuracy in special quantification, and full slides may be preferable to TMA‐based analysis.^22^ The International TILs Working Group recommended analyses of TILs or CD8^+^ T cells using AI‐based approaches for further investigation.^12^, ^23^ Other new technologies such as mass cytometry, single‐cell sequencing, and multicolor IHC are being optimized to assess the level and role of different immune cell types in TME.

Expression of immune checkpoints PD‐1 and TIM3 in T cells involved in tumors often correlates with a state of T‐cell exhaustion. Yet, PD1^+^CD8^+^ TILs in human breast tumors has been reported to remain functional with high cytokine production and degranulation capacity.^24^ TIM3 is also an inhibitory checkpoint that contributes to the immune suppressive microenvironment and can be a resistance mechanism to immunotherapy. Thus PD1^+^ or TIM3^+^ TILs may be predictive or prognostic in BC. In this study, simultaneous testing of CD4, CD8, PD1, TIM3 and cytokeratins were conducted by multicolor IHC and software‐assisted imaging analysis. We investigated the immune checkpoints PD1 and TIM3 expression on CD4^+^ and CD8^+^ T cells in the tumor microenvironment correlated with patient response to NAT.

## Methods

### Patient selection and tumor specimens

A prospective cohort of 50 cases were included for multicolor IHC from June 2017 to December 2018. Inclusion criteria were stage IIB to IIIC BC patients, receiving neoadjuvant treatment (NAT) with informed consent of biomarker testing. For CD3 single‐plex test, a total of 106 nonmetastatic patients including an extra 56 cases between January 2014 and June 2017 at the Affiliated Cancer Hospital & Institute of Guangzhou Medical University were entered into this study. Six patients were excluded because of incomplete clinical information or invalid biomarker data. The NAT involved chemotherapy alone or in combination with trastuzumab. All BC patients were preoperatively treated with the TEC or EC followed by T or EC followed by TH (T: docetaxel/liposome paclitaxel, E: pirarubicin/epirubicin, C: cyclophosphamide, H: trastuzumab) for 2–8 cycles.

Formalin‐fixed and paraffin‐embedded (FFPE) tissue samples were collected both at the time points of diagnosis and surgery. Preneoadjuvant treatment (pre‐NAT) samples were obtained by core needle biopsy of the breast. Surgically‐resected specimens were used as paired post‐NAT samples. In the prospective cohort (*n* = 50), fluorescent multiplex immunohistochemistry (mIHC) was used to stain CD4, CD8, PD‐1, TIM3, and cytokeratins simultaneously. PD1^+^ and TIM3^+^ T cell subsets on full slides were quantified using software‐based methods. Singleplex IHC was also conducted to stain for CD3. Cell levels were correlated with clinicopathological parameters and clinical endpoint pCR.

The study was approved by the ethics review committee of our institution. Written informed consent was obtained from all patients that underwent clinical treatment and biomarker testing. The median follow‐up time for clinical outcome was 2.9 years. Clinicopathological parameters including age, menopausal status, nuclear grade, histologic grade, histologic type, recurrence, follow‐up status, and follow‐up period were obtained by a thorough review of clinical records.

### Clinical molecular typing and pathological response evaluation

To evaluate the molecular subtype classification, the results of immunohistochemistry (IHC) for estrogen receptor (ER), progesterone receptor (PR), and Ki‐67 were reviewed. HER2 expression was assessed by IHC and scoring was determined according to the criteria of American Society of Clinical Oncology (ASCO)/College of American Pathologist (CAP) guidelines. Tumors with scores 2+ were further tested by fluorescence in situ hybridization (FISH). The level of Ki‐67 expression was classified as high versus low with a cutoff point of 20%. ypTN stage was defined according to the American Joint Committee on Cancer. For this study, pCR was defined as the absence of residual invasive cancer in the breast and axillary nodes with the presence or absence of in situ cancer (ypT0/isypN0 or ypT0ypN0), as previously described.^25^

### Histopathologic evaluation of tumor sections by light microscopy

Surgical specimens were dissected, and tissues 0.5 cm thick were used for FFPE block preparation; a total of 696 FFPE blocks were collected, while 1–3 pre‐NAT biopsy samples were obtained from each patient. Tumor sections sliced from FFPE blocks were subjected to hematoxylin and eosin (H&E) staining. Tumor cells and infiltrating lymphocytes were routinely reviewed by two pathologists. Sections with the highest cancer cell content were then subjected to CD3 staining and multiplex IHC.

The number of sections examined for each patient ranged between three and 30, with an average of 5.4 per case. The percentage of viable tumor cells (averaged across all sections) was recorded for each patient, as reported previously.^26^, ^27^ Herein, intratumoral TILs or T cells are defined as lymphocytes or T cells in tumor cell aggregates having cell‐to‐cell contact with no intervening stroma and directly interacting with carcinoma cells, while stromal TILs are located dispersed in the stroma between the carcinoma cells and do not directly contact carcinoma cells.^12^

### Multiplex immunofluorescence staining for CD4, CD8, PD1, TIM3 and cytokeratins and AI‐assisted analyses

**Figure 1:**
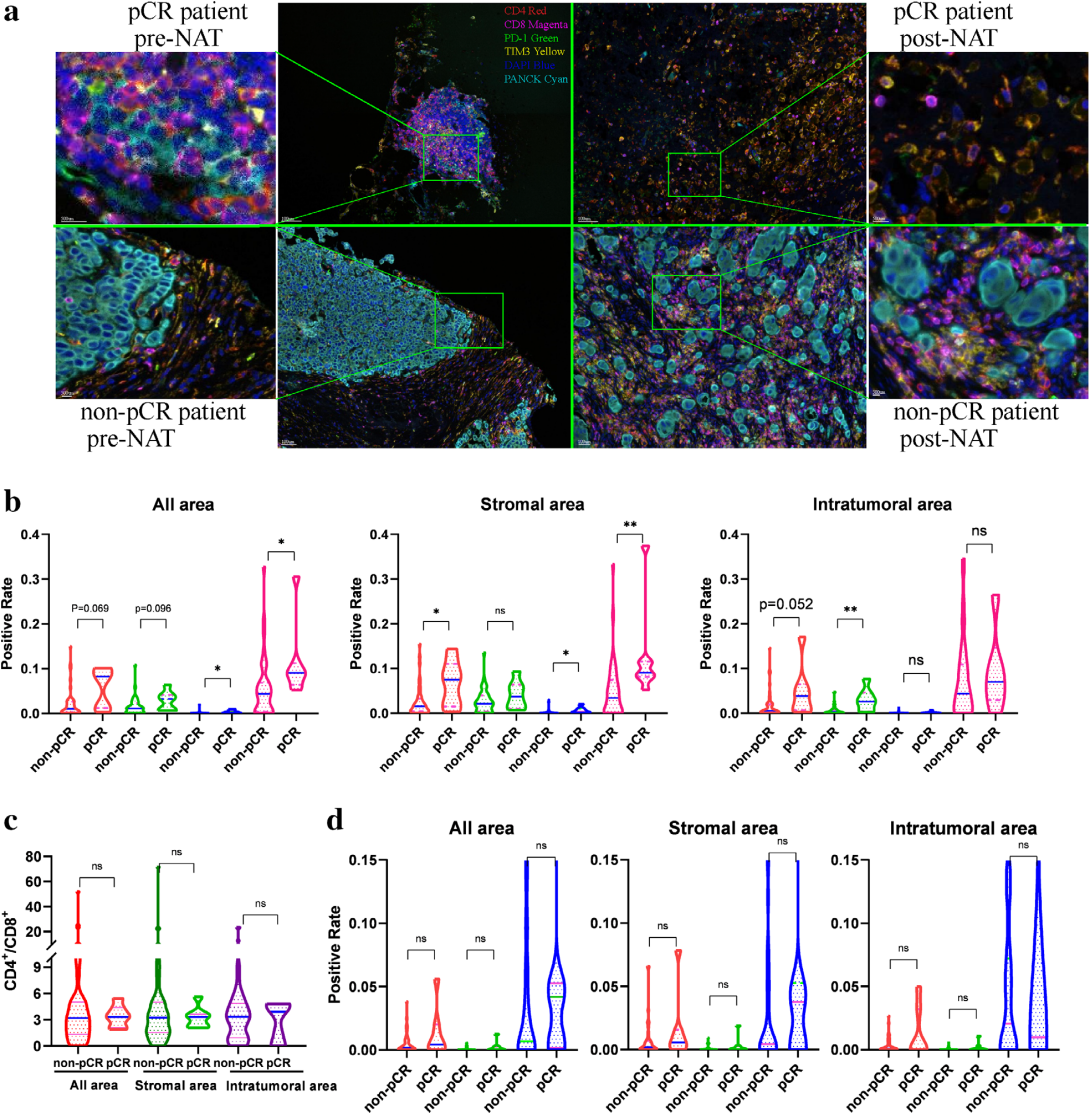
T cell subsets in tumor microenvironment (TME) by mIHC in pre‐NAT tumors. (**a**) Representative mIHC images of preneoadjuvant (left) and post‐neoadjuvant treatment (right) in one pCR patient (top) and one non‐pCR patient (bottom). CD4 (red), CD8 (magenta), PD1 (green), TIM3 (yellow), and CKs (cyan) were stained by fluorescent multiplex IHC, and the percentages of different immune cell subtypes were determined by AI‐assisted methods. (b) The percentages of infiltrating CD8^+^, PD^+^ and PD1^+^CD8^+^ cells, and the ratio of PD1^+^CD8^+^/CD8^+^ in the whole tumor were higher in preneoadjuvant specimens from pCR patients compared to non‐pCR patients (*P* = 0.096, *P* = 0.069, *P* = 0.013, and *P* = 0.012, respectively). The percentages of infiltrating CD8^+^, PD^+^ and PD1^+^CD8^+^ cells, and the ratio of PD1^+^CD8^+^/CD8^+^ also increased in the stroma (*P* = 0.203, *P* = 0.037, *P* = 0.011, and *P* = 0.003) and the intratumoral region (*P* = 0.005, *P* = 0.052, *P* = 0.426, and *P* = 0.582). (**c**) The ratio of CD4^+^/CD8^+^ T cells were not significantly different in either the stromal or intratumoral areas between the pCR patients and non‐pCR patients. (**d**) The percentages of TIM3^+^CD4^+^, TIM3^+^CD8^+^ T cells, and the ratio of TIM3^+^CD8^+^/CD8^+^ T cells, were not significantly different in either the stromal or intratumoral areas between the pCR patients and non‐pCR patients. ns, not significant; * *P* < 0.05; ** *P* < 0.01; *** *P* < 0.001; **** *P* < 0.0001. (**b**) (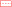) PD^+^, (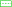) CD8^+^, (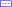) PD1^+^CD8^+^, (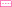) PD1^+^CD8^+^/CD8^+^; (**d**) (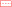) TIM3^+^CD4^+^, (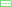) TIM3^+^CD8^+^, (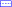) TIM3^+^CD8^+^/CD8^+^

Prospective matched pre‐NAT and post‐NAT tumor samples were subjected to fluorescent multiplex IHC (Fig 1a). Tissue sections 4 μm thick were stained using the PANO 7‐plex IHC kit (Cat. #0004100100, Panovue, Beijing, China), enabling the simultaneous visualization of six markers in the same section. Briefly, antigen retrieval was performed with boiling in antigen retrieval solution AR9 (pH 9). Blocking was performed using the antibody blocking solution (Panovue, Cat. #0018001120) for 15 minutes, followed by incubation with the primary antibodies: TIM3 (CST45208, Cell Signaling Technology, Inc., MA, USA; diluted at 200×), CD8A (CST70306, CST; diluted at 200×), CD4 (BX22300130; diluted at 2000×), PD1 (CST43248, CST; diluted at 100×), and PanCK (CST4545, CST; diluted at 400×). The sections were incubated with the primary antibodies for 30 minutes at room temperature. Subsequently, the sections were incubated with anti‐mouse or anti‐rabbit HRP‐conjugated Polymer (Panovue, Cat. #0013001010) at room temperature for 15 minutes, followed by incubation with TSA Opal fluorophores (PPD 520, PPD 540, PPD 570, PPD 620, PPD 650, and PPD 690) for 10 minutes. After each cycle of staining, the antibody‐TSA complex was removed using AR solution (pH 9) and boiling. After staining, all slides were counterstained with DAPI for five minutes and mounted in ProLong Diamond Antifade Mountant (Thermo Fisher).

To obtain multispectral images, the stained slides were scanned using the PerkinElmer Mantra System (or Polaris System, Waltham, Massachusetts, USA). A spectral library required for multispectral unmixing was established using the inForm image software (inForm 2.4.0 PerkinElmer, Waltham, Massachusetts, USA); reconstructed images of each section were obtained using the spectral library. AI‐assisted analyses using inForm software were performed to determine the recognition and levels of CD4^+^, CD8^+^, PD1^+^, TIM3^+^, PD1^+^CD8^+^, TIM3^+^CD8^+^, PD1^+^CD4^+^, TIM3^+^CD4^+^ T cells. Tumor cells were recognized as CK positive with malignant cytomorphology and sometimes confirmed with reference to HE‐stained slides. Clinical endpoints were blind to data collector and statistician before statistical analysis.

### CD3 staining by singleplex IHC and AI‐assisted analyses

IHC staining for CD3 was conducted using the Dako Omnis autostainer. Briefly, sections 4 μm thick were boiled and then subjected to dewaxing, rehydrating, and antigen retrieval, followed by incubation with an anti‐CD3 primary antibody (Dako Omnis, polyclonal rabbit anti‐human, Santa Clara, CA, USA). For signal visualization, the EnVision FLEX^+^ High pH (Link) system was used, following the manufacturer's instructions. The sections were manually mounted in neutral resin for observation under a light microscope. Whole‐slide images were acquired using the light microscope on the Mantra System (PerkinElmer, Waltham, Massachusetts, US). Images were used to quantify the CD3 signal, and the T cell levels was calculated using the inForm automated image analyses software (PerkinElmer, Waltham, Massachusetts, USA). Briefly, CD3^+^ cells and other cells were recognized by machine‐learning‐based classification according to CD3 staining signal and the percentage was calculated.

### Statistical analysis

All statistical analyses were performed using the SPSS 22.0 software. Continuous variables were described as the mean value ± standard deviation. T cell percentages in pre‐NAT and post‐NAT samples were compared using the Wilcoxon test, while the Mann‐Whitney test and Kruskal‐Wallis test was used for independent samples. Receiver operating characteristic (ROC) curves were used to assess the predictive value of individual biomarkers for pCR. A binary regression model was used to identify significant predictive factors against pCR in univariate and multivariate analyses. *P* < 0.05 was considered statistically significant.

## Results

### **CD8** ^**+**^ **and PD1** ^**+**^ **CD8** ^**+**^ **T cell percentages higher in pre‐NAT tumors of pCR versus non‐pCR patients**

Fluorescent multiplex IHC for CD4, CD8, PD1, TIM3 and cytokeratins was conducted (Fig 1a). Seven prospective patients experienced pCR. Demographic and clinicopathological factors are described in Table 1. Briefly, patients had a median age of 48.6 (range, 23–76). A total of 27 patients were ER^+^, 23 were PR^+^, 16 were HER2^+^, and 36 had high Ki67 expression (>20%). All patients had at least two cycles (range: 2–8 cycles) of neoadjuvant therapy (NAT), involving the administration of chemotherapy alone or in combination with trastuzumab.

**Table 1:** Clinicopathological characteristics and CD8^+^, PD1^+^CD8^+^ T cells in pre‐NAT or post‐NAT tissues

			Pre‐NAT				Post‐NAT			
	n	%	Mean CD8^+^ T cells (%)	*P*‐value	Mean PD1^+^CD8^+^ T cells (%)	*P*‐value	Mean CD8^+^ T cells (%)	*P*‐value	Mean PD1^+^CD8 T cells (%)^+^	*P*‐value
Age				0.922		0.499		0.215		0.787
<35	8	16	1.84		0.11		1.70		0.17	
35–50	22	44	1.82		1 0.21		2.97		0.18	
>50	20	40	1.61		0.21		2.65		0.13	
Menopause status				0.454		0.194		0.197		0.400
Yes	16	32	1.93		0.11		1.97		0.08	
No	34	68	1.95		0.24		2.79		0.19	
Family tumor history				0.586		0.921		0.106		0.578
Yes	2	4	1.74		0.04		4.90		0.10	
No	48	96	1.74		0.20		2.43		0.16	
Number of chemotherapy cycles				0.134		**0.022**		0.705		0.421
2	12	24	1.32		0.01		2.32		0.08	
4	12	24	2.19		0.27		3.18		0.31	
6	20	40	2.14		0.27		2.17		0.13	
8	6	12	0.31		0.01		2.78		0.09	
Clinical response				0.719		0.546		**0.037**		0.953
CR	4	8	2.78		0.20		1.04		0.08	
PR	38	76	1.83		0.23		2.94		0.18	
SD	5	10	1.37		0.10		0.81		0.10	
PD	3	6	0.55		0.01		1.60		0.04	
MPR				0.304		0.337		0.802		**0.059**
Yes	15	30	2.19		0.24		2.46		0.08	
No	35	70	1.58		0.18		2.56		0.19	
pCR				**0.096**		**0.013**		1.000		0.246
Yes	7	14	3.15		0.42		1.75		0.05	
No	43	86	1.53		0.16		2.66		0.18	
Molecular subtype				0.802		0.970		0.719		0.117
Luminal A	4	8	1.58		0.25		2.10		0.03	
Luminal B	25	50	2.09		0.27		2.30		0.08	
HER2 (+)	13	26	1.25		0.08		2.63		0.22	
TNBC	8	16	1.69		0.18		3.22		0.34	
TNM stage				0.935		0.846		0.070		**0.059**
IIB	11	22	2.03		0.27		4.60		0.37	
IIIA	26	52	1.45		0.11		2.30		0.14	
IIIB	1	2	1.01		0.07		1.61		0.00	
IIIC	12	24	2.22		0.35		1.49		0.06	
Total	50	100	1.71		0.19		2.54		0.18	

Clinical response was evaluated according to Response Evaluation Criteria in Solid Tumors (RECIST) 1.1.

CR, complete response; ER, estrogen receptor; HER2, human epidermal growth factor receptor 2; MPR, 10% or less residual viable tumor after neoadjuvant therapy; NAT, neoadjuvant treatment; PD, progressive disease; PR, partial response; PR, progesterone receptor; SD, stable disease; TNBC, triple negative breast cancer.

The percentages of infiltrating CD8^+^, PD1^+^, and PD1^+^CD8^+^ T cells, as well as the ratio of PD1^+^CD8^+^/CD8^+^ T cells in the whole tumor, were marginally or statistically higher in preneoadjuvant therapy (pre‐NAT) samples from pCR patients than in non‐pCR patients (*P* = 0.096, *P* = 0.069, *P* = 0.013, and *P* = 0.012, respectively) (Table 1). Notably, infiltration of T cell subsets was not significantly related to molecular subtypes in both pre‐NAT and post‐NAT samples.

To further look at the spatial distribution of T cells in tumor microenvironment, metrics of T cells in stromal area and intratumoral area were calculated separately. The percentages of CD8^+^, PD1^+^, and PD1^+^CD8^+^ T cells, and the ratio of PD1^+^CD8^+^/CD8^+^ T cells, were also higher at different degrees in both the stromal area (*P* = 0.203, *P* = 0.037, *P* = 0.011, and *P* = 0.003, respectively) and intratumoral area (*P* = 0.005, *P* = 0.052, *P* = 0.426, and *P* = 0.582, respectively) of the pCR patients than in the non‐pCR patients (Fig 1b).

However, the percentages of TIM3^+^CD4^+^, TIM3^+^CD8^+^ T cells, and the ratios of TIM3^+^CD8^+^/CD8^+^ and CD4^+^/CD8^+^ T cells, were not significantly different in either of the stromal or intratumoral areas between the pCR patients and non‐pCR patients (Fig 1c,d).

### Infiltrating CD8 ^+^ and PD1 ^+^ CD8 ^+^ T cell levels predictive of response to NAT

**Figure 2:**
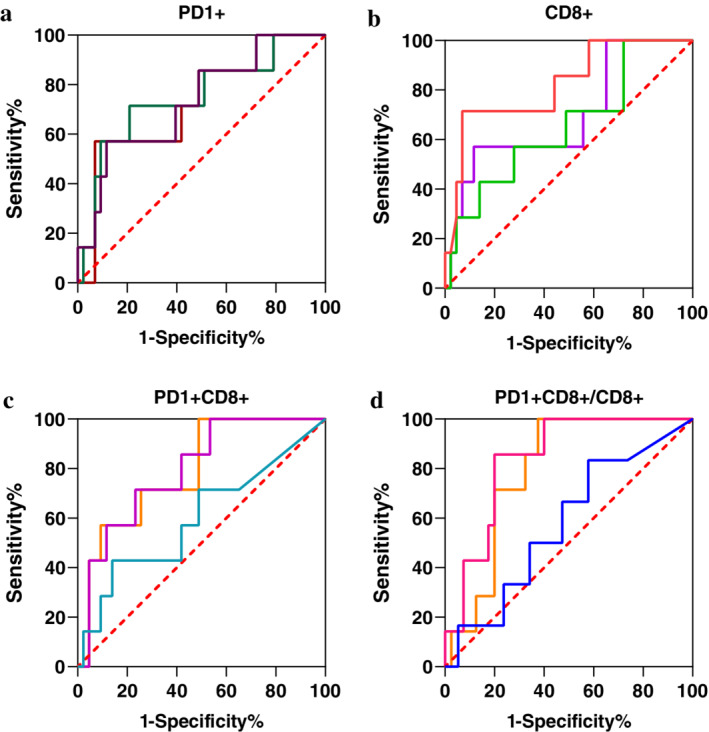
ROC curve analysis of T cell subsets in the tumor microenvironment (TME) in relation to response to NAT. Subsets of PD1^+^ (**a**), CD8^+^ (**b**), PD1^+^CD8^+^ (**c**) T cells, and PD1^+^CD8^+^/CD8^+^ ratio (**d**) were analyzed by receiver operating characteristics (ROC) curve test. The area under the curve for intratumoral CD8^+^ cells, stromal and whole tumor PD1^+^CD8^+^ cells, stromal and whole tumor PD1^+^CD8^+^/CD8^+^ ratio were 0.822 (*P* = 0.007), 0.779 (*P* = 0.013), 0.775 (*P* = 0.014), 0.839 (*P* = 0.005), and 0.793 (*P* = 0.014), respectively. (**a**) (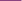) AUC = 0.706, *P* = 0.052, (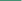) AUC = 0.750, *P* = 0.037, (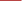) AUC = 0.725, *P* = 0.067; (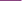) Intratumoral, (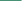) Stromal, (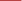) Whole tumor. (**b**) (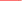) AUC = 0.822, *P* = 0.007, (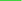) AUC = 0.629, *P* = 0.194, (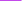) AUC = 0.675, *P* = 0.096; (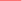) Intratumoral, (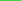) Stromal, (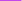) Whole tumor. (**c**) (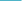) AUC = 0.636, *P* = 0.410, (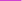) AUC = 0.779, *P* = 0.013, (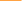) AUC = 0.775, *P* = 0.014; (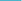) Intratumoral, (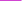) Stromal, (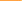) Whole tumor. (**d**) (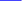) AUC = 0.575, *P* = 0.561, (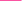) AUC = 0.839, *P* = 0.005, (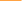) AUC = 0.793, *P* = 0.014; (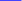) Intratumoral, (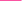) Stromal, (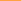) Whole tumor.

The potential use of T cell subsets to predict response to NAT was evaluated by ROC curve analyses. The percentages of PD1^+^, CD8^+^, and PD1^+^CD8^+^ T cells, as well as the PD1^+^CD8^+^/CD8^+^ ratio, in the whole tumor, stromal, and intratumoral area were analyzed (Fig 2a–d). The area under the curve (AUC) values of CD8^+^ T cells levels in the intratumoral area, PD1^+^CD8^+^ T cell levels in the whole tumor and stromal areas, and PD1^+^CD8^+^/CD8^+^ ratio in the whole tumor and stromal areas, were 0.822 (*P* = 0.007), 0.775 (*P* = 0.014), 0.779 (*P* = 0.013), 0.793 (*P* = 0.014), and 0.839 (*P* = 0.005), respectively. Notably, the stromal PD1^+^CD8^+^/CD8^+^ ratio had a sensitivity of 85.7% and specificity of 80.0% at a cutoff of 8.0% (Fig 2d).

To further confirm the predictive value of T cells in BC patients receiving neoadjuvant therapy, binary logistic regression analyses were conducted using data from mIHC‐based immune profiling (50 patients). The stromal PD1^+^CD8^+^/CD8^+^ ratio, the percentage of intratumoral CD8^+^ T cells were significant variables predicting response, both in the univariate and multivariate regression models (Table 2).

**Table 2:** Univariate and multivariate logistic regression for the immune cell subsets and clinical covariates against pCR

		Univariate			Multivariate		
Variable	Categories	Odds ratio	95% CI	*P*‐value	Odds ratio	95% CI	*P*‐value
PD1_All	Continuous	0.025	1.022–1.506	0.029	/	/	/
PD1_Stromal	Continuous	1.229	1.030–1.466	0.022	/	/	/
CD8^+^PD1^+^/CD8^+^_All	Continuous	1.098	0.998–1.207	0.071	/	/	/
CD8^+^PD1^+^/CD8^+^_Stromal	Continuous	1.109	1.009–1.218	0.032	**1.109**	**1.009–1.218**	**0.032**
CD8_Intratumoral	Continuous	1.712	1.052–2.786	0.030	**1.712**	**1.052–2.786**	**0.030**
Reduction of CD8^+^_All	Continuous	1.431	1.005–2.039	0.047	**1.431**	**1.005–2.039**	**0.047**
HR	Negative, Positive	3.923	0.433–35.530	0.224	/	/	/
AGE	Continuous	0.950	0.882–1.023	0.176	/	/	/
NAT cycles	2–8	0.910	0.614–1.349	0.639	/	/	/

For multivariate logistic regression analyses, variables with *P* < 0.2 (Age, PD1_All or PD1_stromal, CD8_Tumor or CD8^+^PD1^+^/CD8^+^_Stromal or CD8^+^PD1^+^/CD8^+^_All) were used.

HR, hormone receptor; NAT, neoadjuvant treatment.

### **Incremental CD8^+^ and PD1** ^**+**^ **CD8** ^**+**^ **T cell levels in non‐pCR tumors after NAT**

**Figure 3:**
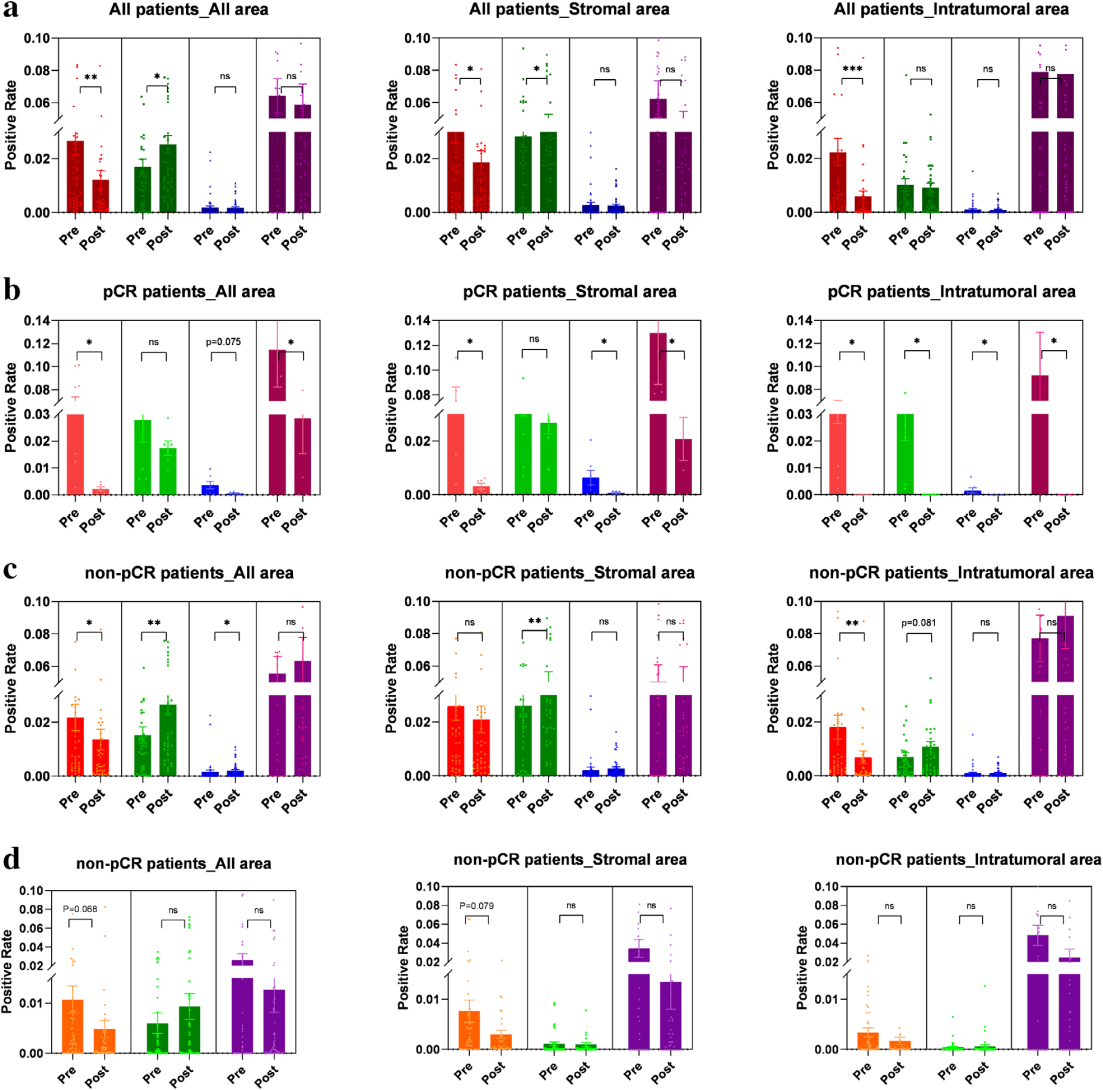
Dynamic changes of percentages of CD8^+^, PD1^+^, PD1^+^CD8^+^ T cell subsets, and the PD1^+^CD8^+^/CD8^+^ ratio in post‐NAT tumors compared with self‐paired pre‐NAT tumors. (**a**) Comparison between paired pre‐NAT and post‐NAT samples of all patients. The percentages of PD1^+^ cells in the whole tumor, stromal, and intratumoral areas significantly decreased post‐NAT (*P* = 0.004, *P* = 0.037, and *P* < 0.001), yet the percentages of CD8^+^ T cells increased (*P* = 0.038, *P* = 0.019, and *P* = 0.832). (**b**) Comparison between paired pre‐NAT and post‐NAT samples of pCR patients. The percentages of PD1^+^, CD8^+^, and PD1^+^CD8^+^ T cells, as well as the PD1^+^CD8^+^/CD8^+^ ratio, decreased in the whole tumor (*P* = 0.028, *P* = 0.173, *P* = 0.075, and *P* = 0.028) and in stromal area (*P* = 0.028, *P* = 0.249, *P* = 0.028, and *P* = 0.028). (**c**) Comparison between paired pre‐NAT and post‐NAT samples of non‐pCR patients. CD8^+^ T cells in all parts of the tumor (*P* = 0.005, *P* = 0.004, *P* = 0.081) and PD1^+^CD8^+^ T cells in the whole tumor area (*P* = 0.047) increased. The percentages of PD1^+^CD8^+^ T cells in the stroma and intratumoral area (*P* = 0.105, and *P* = 0.554, respectively) and the ratio of PD1^+^CD8^+^/CD8^+^ in all parts (*P* = 0.925, *P* = 0.838, and *P* = 0.626, respectively) increased without statistical significance; however, the level of whole area and intratumoral area PD1^+^ cells significantly decreased post‐NAT (*P* = 0.041, *P* = 0.009). (**d**) The percentages of TIM3^+^CD4^+^, TIM3^+^CD8^+^ T cells, and the ratio of TIM3^+^CD8^+^/CD8^+^ T cells, were not significantly increased in either the stromal or intratumoral areas after NAT in non‐pCR patients. Pre, pre‐NAT; Post, post‐NAT. (**a**) (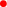) PD1^+^ (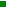) CD8^+^ (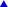) PD1^+^CD8^+^ (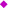) PD1^+^CD8^+^/CD8^+^. (**b**) (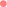) PD1^+^ (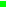) CD8^+^ (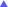) PD1^+^CD8^+^ (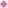) PD1^+^CD8^+^/CD8^+^. (**c**) (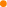) PD1^+^ (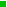) CD8^+^ (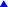) PD1^+^CD8^+^ (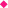) PD1^+^CD8^+^/CD8^+^. (**d**) (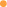) TIM3^+^CD4^+^ (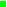) TIM3^+^CD8^+^ (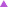) TIM3^+^CD8^+^/CD8^+^.

We also investigated the dynamic changes of infiltrating T cells between self‐paired tumors before and after neoadjuvant chemotherapy. Overall, compared to the pre‐NAT tumors, the percentages of PD1^+^ cells in the whole tumor, stromal, and intratumoral areas significantly decreased post‐NAT (*P* = 0.004, *P* = 0.037, and *P* < 0.001, respectively), yet overall the percentages of CD8^+^ T cells increased (*P* = 0.038, *P* = 0.019, and *P* = 0.832, respectively) (Fig 3a). The reduction of CD8^+^ T cells was also significantly associated with pCR in both univariate and multivariate regression models (Table 2). The dynamic changes of CD8^+^ and PD1^+^CD8^+^ T cells, cancer cell proportions, clinical and pathological responses are shown in Fig S1.

With regard to the metrics in tumors with pCR, the percentages of PD1^+^, CD8^+^, and PD1^+^CD8^+^ T cells, as well as the PD1^+^CD8^+^/CD8^+^ ratio, decreased in the whole tumor (*P* = 0.028, *P* = 0.173, *P* = 0.075, and *P* = 0.028, respectively) and in the stromal area (*P* = 0.028, *P* = 0.249, *P* = 0.028, and *P* = 0.028, respectively) (Fig 3b).

By contrast, in non‐pCR patients, CD8^+^ T cells in all parts of the post‐NAT tumor (*P* = 0.005, *P* = 0.004, *P* = 0.081) and PD1^+^CD8^+^ T cells in the whole tumor area (*P* = 0.047) increased in comparison with pre‐NAT tumors. The percentages of PD1^+^CD8^+^ T cells in the stroma and intratumoral area (*P* = 0.105 and *P* = 0.554) and the ratio of PD1^+^CD8^+^/CD8^+^ in all parts (*P* = 0.925, *P* = 0.838, and *P* = 0.626) increased without statistical significance; however, the level of the whole area and intratumoral area PD1^+^ cells significantly decreased post‐NAT (*P* = 0.041, *P* = 0.009) (Fig 3c). The percentages of TIM3^+^CD4^+^, TIM3^+^CD8^+^ T cells, and the ratio of TIM3^+^CD8^+^/CD8^+^ T cells, were not significantly increased in either the stromal and intratumoral areas after NAT (Fig 3d).

### Percentage of CD3 ^+^ T cells associated with response to NAT

**Figure 4:**
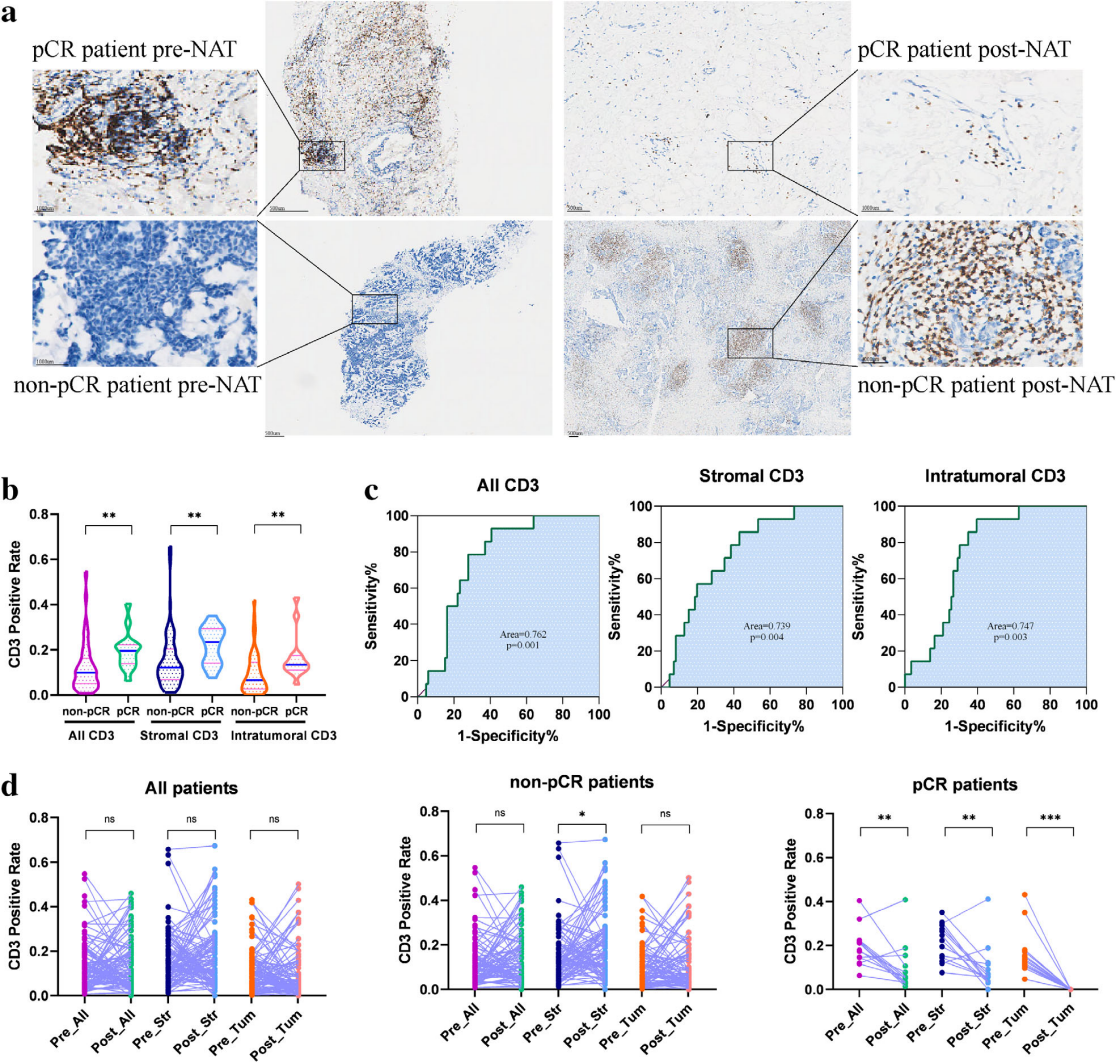
AI‐assisted analyses of CD3^+^ T cells in TME of pre‐NAT and post‐NAT samples. (**a**) Representative CD3^+^ T cells images preneoadjuvant (left) and post‐neoadjuvant treatment (right) in one pCR patient (top) and one non‐pCR patient (bottom). (**b**) The percentage of CD3^+^ T cells was higher in pCR compared to non‐pCR patients in preneoadjuvant therapy specimens. This was the case in the whole tumor (*P* = 0.001), tumor stroma (*P* = 0.004), and intratumoral region (*P* = 0.002). (**c**) Receiver operating characteristics (ROC) curve analyses showed that the area under the curve values for the percentages of CD3^+^ T cells in the whole tumor, stroma, and intratumoral region were 0.762 (*P* = 0.001), 0.739 (*P* = 0.004), and 0.747 (*P* = 0.003), respectively. (**d**) The total levels of CD3^+^ T cells in the whole tumor, stroma, and intratumoral region were not significantly increased after NAT (*P* = 0.795, *P* = 0.169, *P* = 0.401, respectively). In pCR patients, the levels of CD3^+^ T cells decreased significantly (*P* = 0.004, *P* = 0.002, and *P* < 0.001, respectively). In non‐pCR patients, they increased only significantly in the stromal area (*P* = 0.186, *P* = 0.012, *P* = 0.388, respectively). Statistical significance was determined by Wilcoxon rank‐sum test. All_CD3, CD3^+^ T cells in whole tumor; Pre_All, pre‐NAT whole tumor area; Post_All, post‐NAT whole tumor area; Pre_Str, pre‐NAT stromal area; Post_Str, post‐NAT stromal area; Pre_Tum, pre‐NAT intratumoral area; Post_Tum, post‐NAT intratumoral area.

To confirm that T cells are associated with an immediate response to NAT in BC, we also performed a single‐plex IHC staining for the T cell marker CD3 in 100 patients. Whole‐section imaging and AI‐assisted analyses of T cells were conducted (Fig 4a). Demographic and clinicopathological factors are described in Table S1. Briefly, patients had a median age of 49.7 (range, 23–76). A total of 61 patients were ER^+^, 49 were PR^+^, 36 were HER2^+^, and 76 had high Ki67 expression (>20%), and 55 patients (55%) were menopausal.

The percentages of CD3^+^ T cells in the whole tumor, stromal, and intratumoral areas were significantly higher in preneoadjuvant therapy specimens from pCR patients than in non‐pCR patients (*P* = 0.001, *P* = 0.004, *P* = 0.003) (Fig 4b). ROC curve analyses revealed that for the percentages of T cells in the whole tumor, stromal, and intratumoral areas, the area under the curve was 0.762 (*P* = 0.001), 0.739 (*P* = 0.004), and 0.747 (*P* = 0.003), respectively (Fig 4c). The percentages of CD3^+^ T cells in the whole tumor, stromal, and intratumoral areas did not differ significantly between self‐paired pre‐NAT and post‐NAT tumors (*P* = 0.795, *P* = 0.169, *P* = 0.401, respectively). In patients with pCR, the percentages of CD3^+^ T cells decreased in all parts of the tumor with statistical significance (*P* = 0.004, *P* = 0.002, and *P* < 0.001, respectively). In non‐pCR patients, the percentage of CD3^+^ T cells increased; however, this was only significant for the T cells in the tumor stroma (*P* = 0.186, *P* = 0.012, and *P* = 0.388, respectively) (Fig 4d). Binary logistic regression analyses showed the percentage of CD3^+^ T cells in the whole tumor, stromal, and intratumoral areas could predict response, both in the univariate and multivariate models (Table S2).

## Discussion

BC is the most frequently diagnosed cancer and the leading cause of cancer‐related death in women globally.^1^, ^2^, ^3^ NAT is routinely used for high‐risk early‐stage BC to improve the success rate of breast‐conserving surgeries and clinical outcome; NAT has also been reported to be associated with a lower incidence of axillary lymph node metastasis.^28^, ^29^

In breast tumors it is worth focusing on the investigation of the relationship between the levels of different immune cell subtype and clinical outcomes. TILs are very heterogeneous, consisting of CD8^+^ T cells, CD4^+^ helper T cells, regulatory T cells, and B cells, as well as other subtypes of immune cells in the tumor microenvironment.^30^, ^31^ Modern advanced technologies, including single‐cell RNA sequencing, are widely used for tumor immune profiling and have highlighted the heterogeneity in TILs.^32^, ^33^ At present, the predictive and prognostic significance of immune checkpoints on T cells is little understood in the neoadjuvant setting of BC.

Herein, we report in‐depth analyses of T cell levels in a prospective cohort of 50 breast cancer patients. Fluorescent multiplex IHC (mIHC) for simultaneous staining CD4, CD8, PD1, TIM3 and cytokeratins were conducted in paired pre‐NAT and post‐NAT tumor samples. Our analyses involved artificial intelligence (AI)‐based software analyses. To our knowledge, interplay of PD1 and TIM3 positive T cells with neoadjuvant chemotherapy has not yet been investigated in breast tumors.

Our data with AI‐assisted analyses has provided information on cell fraction, density, and distance between particular markers. We found that the fractions of CD3^+^, CD8^+^, and PD1^+^CD8^+^ T cells in stromal and intratumoral regions were significantly well associated with their densities (Fig S2), suggesting that cell percentages and densities could be used exchangeably. Here, we only report the percentage of certain T cell subtypes among the total immune infiltrate. We found that the percentages of infiltrating PD1^+^, CD8^+^ and PD1^+^CD8^+^ T cells, as well as the ratio of PD1^+^CD8^+^/CD8^+^ cells, were higher in pre‐NAT tumors from responders in either the stromal or intratumoral area (tumor cell aggregates area), while CD4^+^, PD1^+^CD4^+^, TIM3^+^CD4^+^, TIM3^+^CD8^+^ cells and CD4^+^/CD8^+^ ratio were not significantly different between pCR and non‐pCR tumors.

ROC curve analyses confirmed the predictive value of the percentages of intratumoral CD8^+^ cells, stromal and whole‐tumor PD1^+^CD8^+^ cell levels, as well as the stromal and whole‐tumor PD1^+^CD8^+^/CD8^+^ ratio. However, the percentages of TIM3^+^CD8^+^ or TIM3^+^CD4^+^ cells could not predict the response to NAT. Our results highlight the potential use of tumor immune cell profiling by multiplex technologies to predict response to therapies. Our findings also suggest that CD8^+^ cytotoxic T lymphocytes (CTLs) or PD1^+^CD8^+^ T cells might have important roles in reshaping the TME after neoadjuvant therapy. PD1^+^ T cells represent exhausted T cells.^34^, ^35^ The association between the percentage of PD1^+^CD8^+^ cells and pCR might result from the fact that PD1 expression is induced during the activation of CD8^+^ T cells.^23^

Both CD4^+^ or CD8^+^ T cell levels have been previously reported to be associated with a response to neoadjuvant therapy of BC in previous studies^18，19^, although with contradictory results between individual studies. In our study, we found that CD8^+^ and PD1^+^CD8^+^ T cell levels correlated with pCR, suggesting PD1^+^CD8^+^ cells may be functional in anticancer immunity. The upregulation of PD1 and other negative receptors has been linked to adaptive resistance to immunotherapy and other treatments.^35^ However, PD1^+^CD8^+^ T cells have been recently recognized to be actively multifunctional with cytokine production and degranulation activity, especially in breast cancer.^24^ This suggests that these PD1^+^CD8^+^ T cells in BC could be harnessed for effective immunotherapies. Thus, in a neoadjuvant setting, our data indicated PD1^+^CD8^+^ T cells might be functionally related with pCR.

It has been previously reported that pCR in breast cancer is associated with molecular subtypes in large sample size analysis.^6^ TILs have been reported to be enriched in HER2+ and TNBC tumors^11^, ^13^ suggesting that molecular subtype may confound the predictive role of T cell subsets. In our study, using Fisher's exact test, the molecular subtypes of breast cancer were determined not to be related to the response of neoadjuvant chemotherapy. T cell subsets were also not associated with molecular subtype as shown in Table 1. Thus we infer that PD1^+^ CD8^+^ and CD8^+^ T cell subsets in pre‐NAT tumors may have the capacity to predict treatment response in all molecular subtypes of BC.

To confirm if total T cells were associated with immediate response to NAT in this study, we also performed single‐plex IHC staining for the T cell marker CD3 in the prospective 50 cases and an additional retrospective cohort of 50 patients. Our results showed AI‐based analyses (Fig S3) of CD3^+^ T cell percentages could predict pathological response to NAT. These results are in line with a previously reported association between pre‐NAT TIL levels and pCR.^11^, ^13^, ^14^, ^15^ Yet, this single‐plex IHC data alone does not confirm the expression status of immune checkpoint molecules such as PD1 and TIM3.

Our study further provides insight into the spatial distribution of CD4^+^, CD8^+^, PD1^+^CD8^+^, PD1^+^CD4^+^, TIM3^+^CD8^+^, and TIM3^+^CD4^+^ cells within different areas of the tumor (Table S3). In pCR patients, the difference of the cell distribution between the stroma and tumor core were not significant. T cell infiltration into, and contact with, the tumor core may predispose these patients to pCR, but in non‐pCR patients, we found that several of the T cell subsets, including CD4^+^, CD8^+^, PD^+^CD4^+^, PD1^+^CD8^+^, TIM3^+^CD4^+^, TIM3^+^CD8^+^ cells, were more abundant in the tumor stroma than in the intratumoral area of the tumor (Table S3). This enrichment of T cells in the stromal area was also demonstrated by CD3 staining. This finding is in line with distribution of TILs in other studies involving NAT of BC patients.^13^ This might be explained by the possibility that tumor cells have the ability to suppress the infiltration of T cells through mechanisms that are yet to be elucidated. In future, how to compromise the ability of cancer cells to achieve more T cell infiltration into the tumor core could be one research direction to improve anticancer treatment.

We also assessed the dynamic changes in the immune cell composition in TME after NAT, by comparing paired pre‐NAT and post‐NAT samples (ie, before and after neoadjuvant treatment). Previous reports also showed the frequency of CD3^+^, and CD8^+^ cells decreased in pCR patients.^11^, ^13^, ^16^ Here, we report new data that the frequency of PD1^+^ and PD1^+^CD8^+^ cells, as well as the ratio of PD1^+^CD8^+^/CD8^+^ cells, also decreased in pCR patients. Moreover, the frequency of T cells was significantly higher in post‐neoadjuvant samples from non‐pCR patients compared to pCR patients (Fig S4a), suggesting that before NAT the enrichment of competent immune T cells might be more important to tumor cell elimination.

Interestingly in non‐pCR patients, the levels of tumor‐infiltrating CD8^+^ and PD1^+^CD8^+^ cells, as well as the PD1^+^CD8^+^/CD8^+^ ratio, increased after NAT, while the percentage of PD1^+^ cells significantly decreased. This observation was contrary to the great decrease of T cells in pCR tumors after NAT. This dynamic data suggests that the changes in the percentages of PD1^+^ and PD1^+^CD8^+^ cells, rather than TIM3^+^ or TIM3^+^CD8^+^, might be one of the mechanisms by which NAT regulates TME. We also found T cells remained higher in post‐NAT tumor tissues compared with adjacent nontumor tissues (Fig S4b). We therefore speculate that in non‐pCR patients the special increase in the percentages of PD1^+^CD8^+^ and CD8^+^ cells after NAT suggest a rationale for the combination of chemotherapy with PD1 inhibitors in the neoadjuvant setting.

Taken together, our data showed that the levels of all, or some, special subsets of T cells could predict pCR to neoadjuvant therapy in BC patients. AI‐based analysis of multiple markers in full slides is feasible; however, it requires further validation and standardization to be translated into clinical use. Whole tissue slides would be preferable for further validation as significant variations among assessment methods have been reported to occur in tissue microarray‐based TIL analysis.^22^ Interaction and linearity tests of T cells should be included in future studies to determine and validate biomarker thresholds.

This study had several limitations. It was a single‐center study, and we did not assess the relationship between immune markers and PFS or OS due to the lack of sufficient clinical information. In addition, we did not include data on the levels of TILs during the interval of NAT, as on‐treatment biopsy samples were not available. Our analyses were restricted to CD4, CD8, PD1, and TIM3; several other markers of critical immune cell subtypes have been established, including FoxP3, CD127, CD68, CD103, and CD19, but these were not examined due to the limited number of markers that can be stained in the same run using mIHC and shortage of sufficient pre‐NAT small biopsy samples.

In conclusion, our results demonstrated that simultaneous quantification of immune checkpoints and T cell markers in clinical samples are feasible. PD1^+^CD8^+^ rather than TIM3^+^CD8^+^ cells are the main subsets of infiltrating T cells to predict response to neoadjuvant chemotherapy in BC. Multiplex biomarker panel analysis can provide the exact identity of those cells stained by fluorescence in comparison to morphological assessment. Software‐assisted analyses may overcome the shortcomings of other TIL assessment approaches in terms of reproducibility and objectivity. The dynamically incremental percentages of PD1^+^CD8^+^ cells in non‐pCR patients' tumors also suggest a potential benefit of the combination of chemotherapy and PD1 inhibition in a neoadjuvant setting. However, this merits further investigation.

## Disclosure

All authors declare that they have no conflicts of interest.

## Supporting information


**Figure S1** Association between the percentage of CD8^+^, PD1^+^CD8^+^ T cells in pre‐NAT or post‐NAT tissues, change in T cells, molecular subtype, and response to neoadjuvant therapy. Observations are ranked by the pre‐NAT CD8^+^ T cell percentages. Patients with pCR are more frequently among those with a high percentage of tumor infiltrating lymphocytes as shown at the lower right side of the figure (gray bar). NAT, neoadjuvant therapy; pCR, pathological complete response; non‐pCR, nonpathological complete response; RECIST1.1, the response evaluation criteria in solid tumors; CR, complete response; PR, partial response; SD, stable disease; PD, progressive disease.

**Figure S2** The correlation between different characteristics of the T cells in the tumor microenvironment. The Pearson correlation test for the percentages of PD1^+^, CD4^+^, CD8^+^, TIM3^+^, CD4^+^ CD8^+^, CD3^+^ cells and densities of PD1^+^, CD4^+^, CD8^+^, TIM3^+^ cells calculated as number of cells per square millimeter. PosRate, positive rate; Dens, density.

**Figure S3** Recognition of T cells by AI‐assisted analysis of CD3 in IHC images. CD3^+^ cells were labeled with brown color, and non‐CD3^+^ cells were labeled with blue color. In AI‐based analyses, CD3^+^ cells and other cells were recognized by machine‐learning‐based classification according to CD3 staining signal and the percentage was calculated.

**Figure S4** Comparison of the percentage of TILs in post‐NAT tissues between non‐pCR and pCR patients, as well as between post‐NAT tumors and adjacent tissues of non‐pCR patients. (**a**) The percentage of TILs was significantly higher in post‐NAT specimens from non‐pCR patients compared with pCR patients. (**b**) The percentage of TILs was significantly higher in the tumor compared to the adjacent nontumor tissue in post‐NAT specimens of non‐pCR patients. **** *P* < 0.0001

**Table S1** Clinicopathological characteristics and CD3^+^ T cells in pre‐NAT or post‐NAT tissues (N = 100).

**Table S2** Univariate and multivariate logistic regression analyses of CD3^+^ T cells and clinical covariates against pCR.

**Table S3** Comparisons of the percentages of immune cell subsets between the stromal and intratumoral areas in pre‐NAT or post‐NAT tissue specimens (Wilcoxon test).
