# Supplementary figures and images for: Quantitative multiplex immunofluorescence analysis identifies infiltrating PD1+CD8+ and CD8+ T cells as predictive of response to neoadjuvant chemotherapy in breast cancer
Source: Thorac Cancer. 2020 Sep 7;11(10):2941–54. doi: 10.1111/1759-7714.13639 (PMC7529566; doi:10.1111/1759-7714.13639)

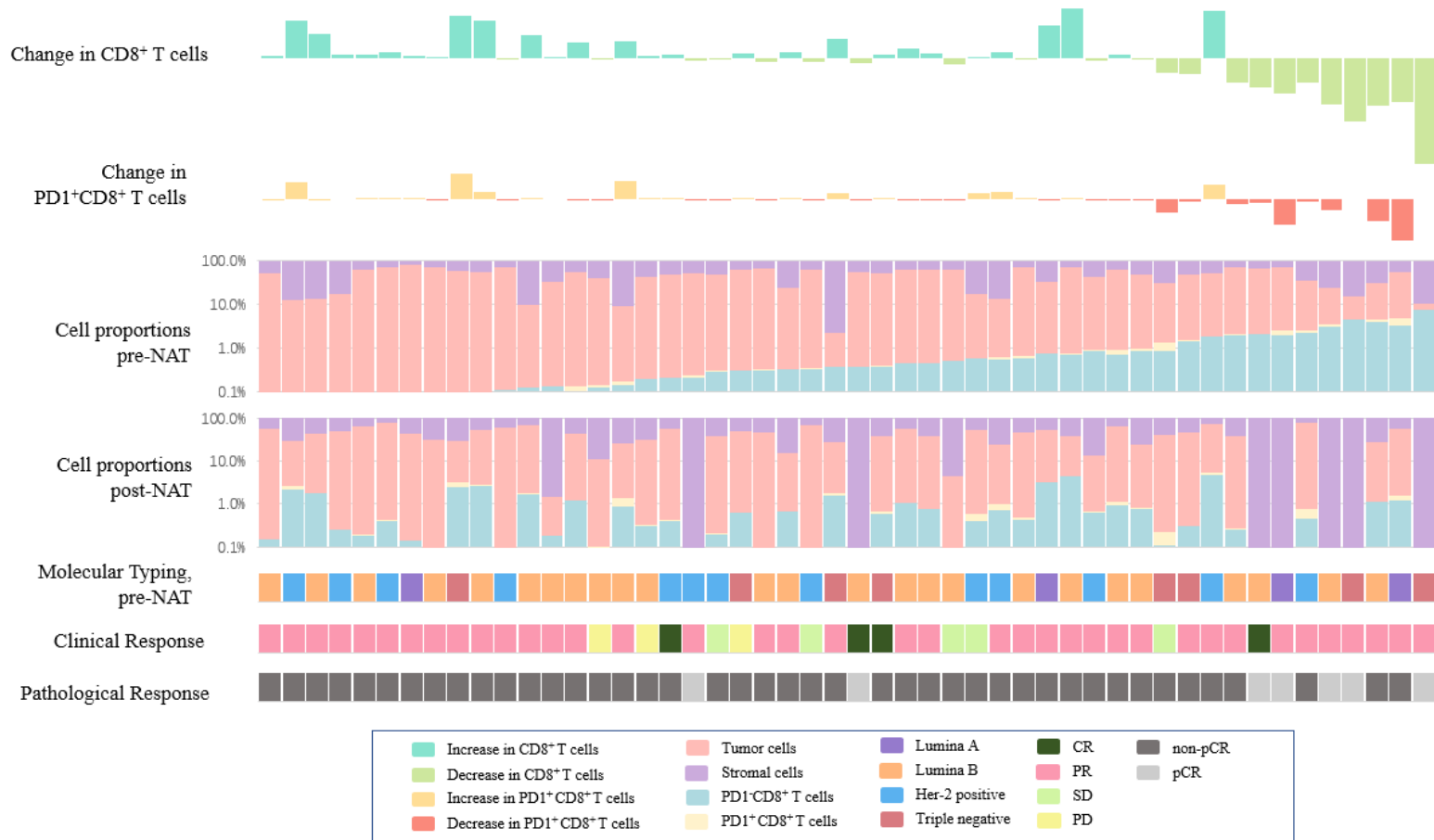

Supplement: Supplementary file 1 — Figure S1 Association between the percentage of CD8+, PD1+CD8+ T cells in pre‐NAT or post‐NAT tissues, change in T cells, molecular subtype, and response to neoadjuvant therapy. Observations are ranked by the pre‐NAT CD8+ T cell percentages. Patients with pCR are more frequently among those with a high percentage of tumor infiltrating lymphocytes as shown at the lower right side of the figure (gray bar). NAT, neoadjuvant therapy; pCR, pathological complete response; non‐pCR, nonpathological complete response; RECIST1.1, the response evaluation criteria in solid tumors; CR, complete response; PR, partial response; SD, stable disease; PD, progressive disease. [file TCA-11-2941-s001.pdf]

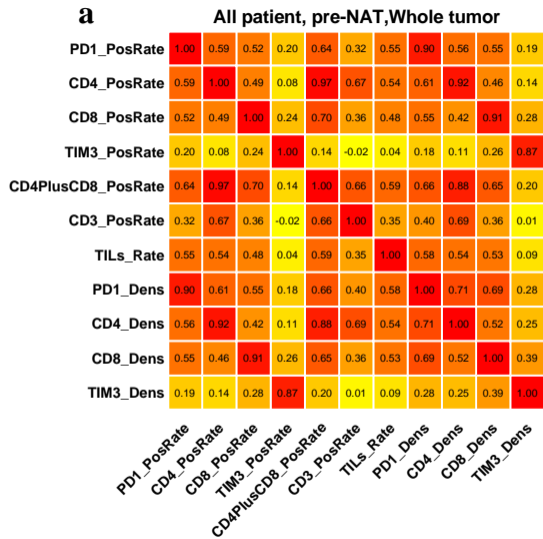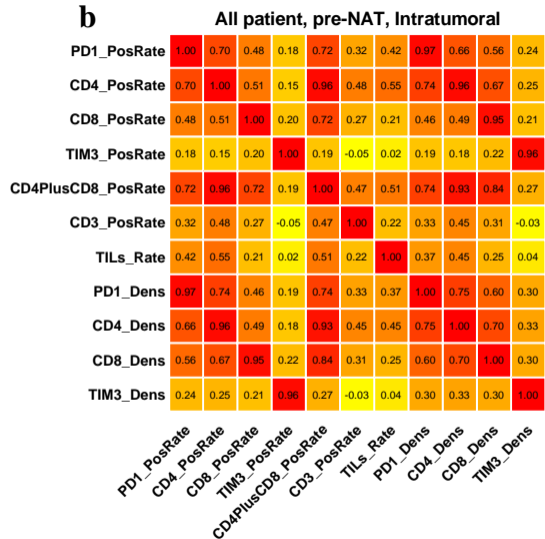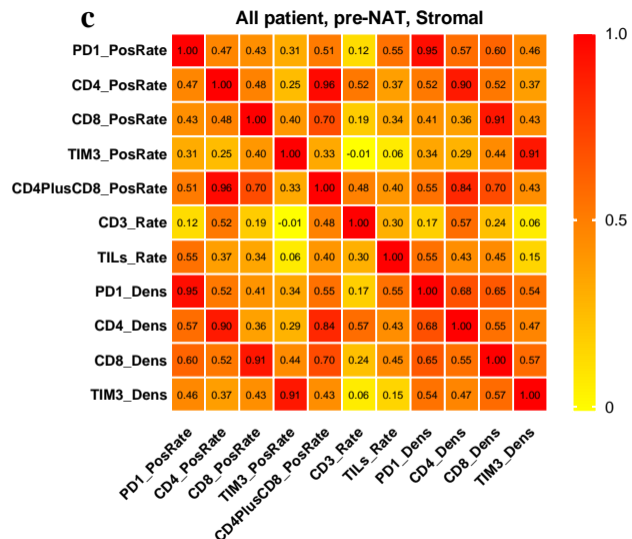

Supplement: Supplementary file 2 — Figure S2 The correlation between different characteristics of the T cells in the tumor microenvironment. The Pearson correlation test for the percentages of PD1+, CD4+, CD8+, TIM3+, CD4+ CD8+, CD3+ cells and densities of PD1+, CD4+, CD8+, TIM3+ cells calculated as number of cells per square millimeter. PosRate, positive rate; Dens, density. [file TCA-11-2941-s002.pdf]

# 66133  
pre-NAT  
non-pCR

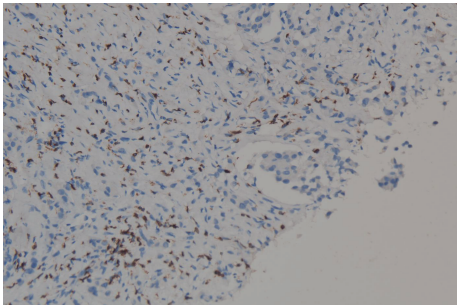

CD3 IHC Original graph

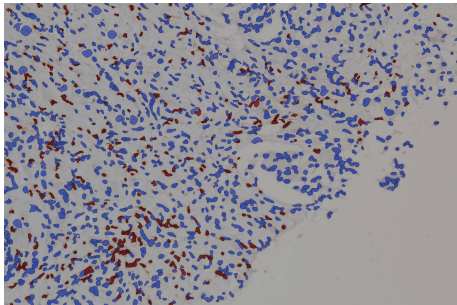

CD3 IHC AI-assisted graph

Supplement: Supplementary file 3 — Figure S3 Recognition of T cells by AI‐assisted analysis of CD3 in IHC images. CD3+ cells were labeled with brown color, and non‐CD3+ cells were labeled with blue color. In AI‐based analyses, CD3+ cells and other cells were recognized by machine‐learning‐based classification according to CD3 staining signal and the percentage was calculated. [file TCA-11-2941-s003.pdf]

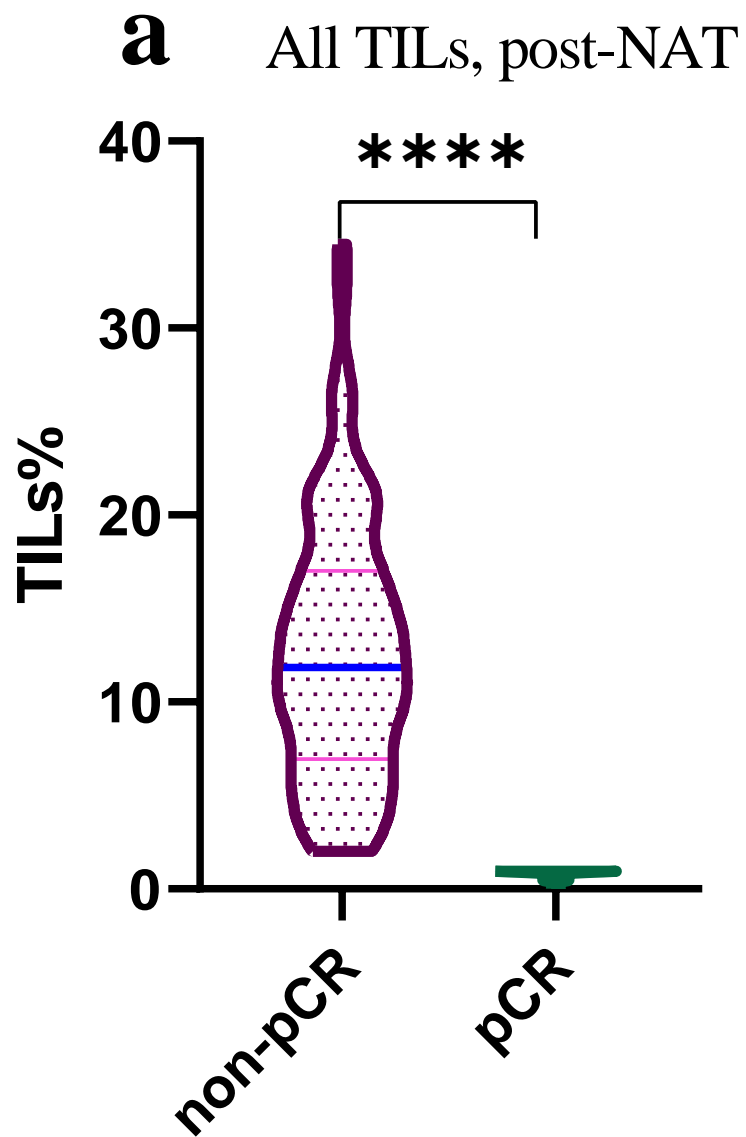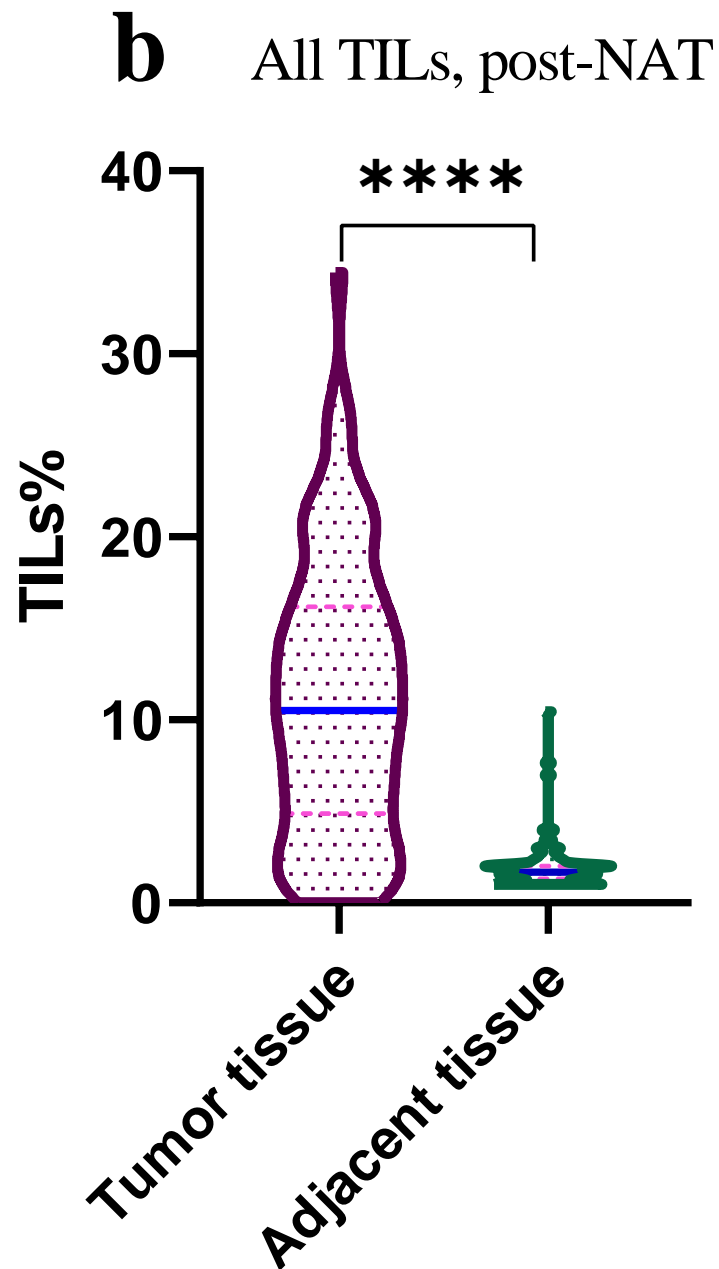

Supplement: Supplementary file 4 — Figure S4 Comparison of the percentage of TILs in post‐NAT tissues between non‐pCR and pCR patients, as well as between post‐NAT tumors and adjacent tissues of non‐pCR patients. (a) The percentage of TILs was significantly higher in post‐NAT specimens from non‐pCR patients compared with pCR patients. (b) The percentage of TILs was significantly higher in the tumor compared to the adjacent nontumor tissue in post‐NAT specimens of non‐pCR patients. **** P < 0.0001 [file TCA-11-2941-s004.pdf]
