# Supplementary material for: Quantitative multiplex immunofluorescence analysis identifies infiltrating PD1+CD8+ and CD8+ T cells as predictive of response to neoadjuvant chemotherapy in breast cancer
Source: Thorac Cancer. 2020 Sep 7;11(10):2941–54. doi: 10.1111/1759-7714.13639 (PMC7529566; doi:10.1111/1759-7714.13639)
Supplement: Supplementary file 5 — Table S1 Clinicopathological characteristics and CD3+ T cells in pre‐NAT or post‐NAT tissues (N = 100). [file TCA-11-2941-s005.doc]

**Supplementary Table 1. Clinicopathological characteristics and CD3^+^ T cells in pre-NAT or post-NAT tissues (N=100).**

|  | **n** | **%** | **Pre-NAT Post-NAT** | | | | |  |
| --- | --- | --- | --- | --- | --- | --- | --- | --- |
|  |  |  | **Mean CD3^+^ T cells(%)** | ***P*** |  | **Mean CD3^+^ T cells (%)** | ***P*** | |
| **Age** |  |  |  | 0.777 |  |  | 0.357 | |
| <35 | 10 | 10.0 | 14.57 |  |  | 12.39 |  | |
| 35-50 | 42 | 42.0 | 3.96 |  |  | 15.81 |  | |
| >50 | 48 | 48.0 | 13.25 |  |  | 12.29 |  | |
| **Menopause**  **Status** | | |  | 0.159 |  |  | 0.141 | |
| Yes | 55 | 55.0 | 12.19 |  |  | 11.51 |  | |
| No | 45 | 45.0 | 14.85 |  |  | 15.61 |  | |
| **Family tumor**  **History** | | |  | 0.539 |  |  | 0.385 | |
| Yes | 7 | 7.0 | 11.72 |  |  | 16.24 |  | |
| No | 93 | 93.0 | 13.84 |  |  | 13.62 |  | |
| **Number of**  **Chemotherapy cycle** | | |  | 0.209 |  |  | 0.403 | |
| 2 | 30 | 30.0 | 13.38 |  |  | 11.64 |  | |
| 4 | 32 | 32.0 | 13.22 |  |  | 14.44 |  | |
| 6 | 30 | 30.0 | 15.53 |  |  | 14.02 |  | |
| 8 | 7 | 7.0 | 9.495 |  |  | 18.92 |  | |
| **Clinical Response** | | |  | 0.857 |  |  | 0.072 | |
| CR | 11 | 11.0 | 14.19 |  |  | 8.23 |  | |
| PR | 66 | 66.0 | 13.25 |  |  | 15.49 |  | |
| SD | 19 | 19.0 | 13.81 |  |  | 11.75 |  | |
| PD | 4 | 4.0 | 18.64 |  |  | 10.18 |  | |
| **MPR** |  |  |  | **0.005** |  |  | 0.132 | |
| Yes | 20 | 20.0 | 18.24 |  |  | 10.63 |  | |
| No | 80 | 80.0 | 12.52 |  |  | 14.62 |  | |
| **pCR** |  |  |  | **0.002** |  |  | **0.041** | |
| Yes | 14 | 14.0 | 19.67 |  |  | 8.41 |  | |
| No | 86 | 86.0 | 12.69 |  |  | 14.71 |  | |
| **ER** |  |  |  | 0.390 |  |  | 0.390 | |
| Negative | 39 | 39.0 | 15.38 |  |  | 14.47 |  | |
| Positive | 61 | 61.0 | 12.57 |  |  | 13.37 |  | |
| **PR** |  |  |  | 0.251 |  |  | 0.826 | |
| Negative | 51 | 51.0 | 15.56 |  |  | 13.81 |  | |
| Positive | 49 | 49.0 | 11.65 |  |  | 13.81 |  | |
| **HER-2** |  |  |  | 0.918 |  |  | **0.051** | |
| Negative | 64 | 64.0 | 13.85 |  |  | 12.09 |  | |
| Positive | 36 | 36.0 | 13.40 |  |  | 16.91 |  | |
| **KI-67** |  |  |  | 0.555 |  |  | 0.869 | |
| Low | 24 | 24.0 | 12.87 |  |  | 15.37 |  | |
| High | 76 | 76.0 | 13.94 |  |  | 13.30 |  | |
| **Disease Stage** | | |  | 0.635 |  |  | 0.855 | |
| IIB | 21 | 21.0 | 13.02 |  |  | 12.80 |  | |
| IIIA | 43 | 43.0 | 15.42 |  |  | 14.78 |  | |
| IIIB | 4 | 4.0 | 16.51 |  |  | 12.24 |  | |
| IIIC | 32 | 32.0 | 11.34 |  |  | 13.37 |  | |
| **Total** | 100 | 100.0 | 13.68 |  |  | 13.81 |  | |

NAT, neo-adjuvant treatment; RECIST, The Response Evaluation Criteria In Solid Tumors; CR, complete response; PR, partial response; SD, stable Disease; PD, progressive disease; MPR, 10% or less residual viable tumor after neoadjuvant therapy; ER, estrogen receptor; PR, progesterone receptor; HER2, human epidermal growth factor receptor 2.
