# Supplementary material for: Quantitative multiplex immunofluorescence analysis identifies infiltrating PD1+CD8+ and CD8+ T cells as predictive of response to neoadjuvant chemotherapy in breast cancer
Source: Thorac Cancer. 2020 Sep 7;11(10):2941–54. doi: 10.1111/1759-7714.13639 (PMC7529566; doi:10.1111/1759-7714.13639)
Supplement: Supplementary file 6 — Table S2 Univariate and multivariate logistic regression analyses of CD3+ T cells and clinical covariates against pCR. [file TCA-11-2941-s006.doc]

**Supplementary Table 2.** Univariate and multivariate logistic regression analyses of CD3^+^ T cells and clinical covariates against pCR.

| **Variable** | **Categories** | **Univariate** | | |  | | **Multivariate** | | | | | |
| --- | --- | --- | --- | --- | --- | --- | --- | --- | --- | --- | --- | --- |
|  |  | Odds ratio | 95% CI | *P* |  | | Odds ratio | | 95% CI | | *P* | |
| **Stromal CD3** | Continuous | **1.039** | **0.998-1.081** | **0.060** |  | 1.039 | | 0.998-1.081 | | 0.060 | |  |
| **Intratumoral CD3** | Continuous | **1.062** | **1.010-1.117** | **0.020** |  | **1.062** | | **1.010-1.117** | | **0.020** | |  |
| **Total CD3** | Continuous | **1.049** | **1.004-1.096** | **0.033** |  | **1.049** | | **1.004-1.096** | | **0.033** | |  |
| **KI-67** | <20% Low,  >20% High | **0.271** | **0.073-1.011** | **0.052** |  | | / | | / | | / | |
| ER | Negative, Positive | 0.322 | 0.460-10.604 | 0.322 |  | | / | | / | | / | |
| Age | Continuous | 0.288 | 0.260-1.493 | 0.288 |  | | / | | / | | / | |
| NAT cycles | 2-8 | 1.034 | 0.769-1.389 | 0.827 |  | | / | | / | | / | |

For multivariate logistic regression analysis, variables with *P* < 0.2 (KI-67, stromal CD3 or intratumoral CD3 or total CD3) were entered. ER，estrogen receptor; NAT, neo-adjuvant treatment.
