# Supplementary material for: Quantitative multiplex immunofluorescence analysis identifies infiltrating PD1+CD8+ and CD8+ T cells as predictive of response to neoadjuvant chemotherapy in breast cancer
Source: Thorac Cancer. 2020 Sep 7;11(10):2941–54. doi: 10.1111/1759-7714.13639 (PMC7529566; doi:10.1111/1759-7714.13639)
Supplement: Supplementary file 7 — Table S3 Comparisons of the percentages of immune cell subsets between the stromal and intratumoral areas in pre‐NAT or post‐NAT tissue specimens (Wilcoxon test). [file TCA-11-2941-s007.doc]

**Supplementary Table 3.** Comparisons of the percentages of immune cell subsets between the stromal and intratumoral areas in pre-NAT or post-NAT tissue specimens (Wilcoxon test).

| **TIL subsets** | **Pre-NAT All patients** | | | **Pre-NAT non-pCR patients** | | | **Pre-NAT pCR patients** | | | **Post-NAT non-pCR patients** | | |
| --- | --- | --- | --- | --- | --- | --- | --- | --- | --- | --- | --- | --- |
|  | **sTILs** | **itTILs** | ***P* value** | **sTILs** | **itTILs** | ***P* value** | **sTILs** | **itTILs** | ***P* value** | **sTILs** | **itTILs** | ***P* value** |
| **PD1^+^** | 0.032 | 0.022 | **0.001** | 0.260 | 0.018 | **<0.0001** | 0.067 | 0.049 | 0.128 | 0.019 | 0.006 | **<0.0001** |
| **CD4^+^** | 0.087 | 0.024 | **<0.0001** | 0.081 | 0.020 | **<0.0001** | 0.123 | 0.050 | **0.018** | 0.066 | 0.013 | **<0.0001** |
| **CD8^+^** | 0.028 | 0.009 | **<0.0001** | 0.026 | 0.007 | **<0.0001** | 0.041 | 0.023 | 0.173 | 0.047 | 0.010 | **<0.0001** |
| **TIM3^+^** | 0.026 | 0.031 | 0.280 | 0.024 | 0.028 | **0.001** | 0.043 | 0.053 | 0.075 | 0.014 | 0.012 | **0.001** |
| **PD1^+^CD8^+^** | 0.003 | 0.001 | **<0.0001** | 0.002 | 0.001 | **<0.0001** | 0.006 | 0.002 | 0.686 | 0.002 | 0.001 | **<0.0001** |
| **TIM3^+^CD8^+^** | 0.001 | 0.001 | **0.007** | 0.001 | 0.000 | **0.002** | 0.003 | 0.002 | 0.715 | 0.0009 | 0.0005 | **0.002** |
| **PD1^+^CD4^+^** | 0.008 | 0.002 | **<0.0001** | 0.006 | 0.001 | **<0.0001** | 0.016 | 0.006 | **0.028** | 0.006 | 0 | **<0.0001** |
| **TIM3^+^CD4^+^** | 0.009 | 0.005 | **0.002** | 0.008 | 0.003 | **<0.0001** | 0.017 | 0.012 | 0.173 | 0.003 | 0.002 | **<0.0001** |
| **PD1^+^CD8^+^/CD8^+^** | 0.065 | 0.079 | 0.474 | 0.053 | 0.077 | **0.007** | 0.143 | 0.092 | 0.715 | 0.047 | 0.088 | **0.007** |
| **TIM3^+^CD8^+^/CD8^+^** | 0.041 | 0.053 | **0.015** | 0.053 | 0.048 | **0.035** | 0.072 | 0.086 | 1.000 | 0.014 | 0.024 | **0.035** |

**sTILs:** **Stromal tumor-infiltrating lymphocytes, itTILs:** **Intratumoral tumor-infiltrating lymphocytes**
